# Novel Ethyl 2-Aminopyrano[3,2-c]isochromene-3-carboxylate Derivatives: Synthesis, Spectral Characterization, and Cytotoxicity

**DOI:** 10.5812/ijpr-168116

**Published:** 2026-06-09

**Authors:** Mehdi Abaszadeh, Fatemeh Haghani, Ehsan Faghihmirzaei, Salehe Sabouri

**Affiliations:** 1 Pharmaceutics Research Center, Institute of Pharmaceutical Sciences, Kerman University of Medical Sciences, Kerman, Iran; 2 Pharmaceutical Sciences and Cosmetic Products Research Center, Institute of Pharmaceutical Sciences, Kerman University of Medical Sciences, Kerman, Iran; 3 Department of Chemistry, University of Warwick, Coventry, United Kingdom; 4 Herbal and Traditional Medicines Research Center, Institute of Pharmaceutical Sciences, Kerman University of Medical Sciences, Kerman, Iran; 5 Department of Pharmaceutical Biotechnology, Faculty of Pharmacy, Kerman University of Medical Sciences, Kerman, Iran

**Keywords:** Anticancer, Antineoplastic, Antiproliferative, Three-component Reaction, 4-hydroxyisocoumarin, Ethyl 2-aminopyrano[3, 2-c]isochromene-3-carboxylate Derivative

## Abstract

**Background:**

Cancer remains one of the most challenging threats to human health and has prompted intensive research in anticancer drug discovery and synthesis. Isocoumarins are natural lactones with several pharmacological activities, including cytotoxic and anticancer effects.

**Objectives:**

This study aimed to synthesize novel ethyl 2-aminopyrano[3,2-c]isochromene-3-carboxylate derivatives and evaluate their cytotoxicity against the MCF-7 breast cancer and A549 lung cancer cell lines, in comparison with a normal cell line (MCF-10A human breast cells).

**Methods:**

First, 4-hydroxyisocoumarin was prepared. A series of ethyl 2-aminopyrano[3,2-c]isochromene-3-carboxylate derivatives was then synthesized via a one-pot, three-component reaction of 4-hydroxyisocoumarin, ethyl cyanoacetate, and aromatic aldehydes in ethanol under reflux in the presence of triethylamine. The resulting compounds were characterized using standard spectroscopic techniques, including IR, ^1^H NMR, and ^13^C NMR, as well as elemental analyses. Finally, the cytotoxicity of the synthesized compounds was evaluated in MCF-7, A549, and MCF-10A cell lines using a colorimetric MTT assay.

**Results:**

The compounds were successfully synthesized, and the cytotoxicity assay demonstrated dose-dependent cytotoxic effects in the tested cell lines. Most compounds exhibited moderate or low toxicity, whereas some were non-toxic in these cells. The most cytotoxic compounds were 4g, 4n, and 4m, with IC_50_ values of 120.77 ± 7.64, 141.43 ± 13.81, and 168.62 ± 3.59 μg/mL against MCF-7 cells, respectively, and 4a, with an IC_50_ value of approximately 131.12 ± 11.00 μg/mL against A549 cells. Compared with MCF-10A non-cancerous cells, these compounds showed selectivity indices (SIs) of 4.14, 1.64, 2.47, and 3.42, respectively.

**Conclusions:**

The compounds were synthesized in high yields and exhibited moderate-to-mild toxicity toward MCF-7, A549, and MCF-10A cells. Notably, halogen substitution at the ortho position of the phenyl ring increased toxicity, particularly in the MCF-7 cell line. However, the lack of selectivity observed for most compounds indicates that further structural refinement is required before this scaffold can be considered a viable anticancer lead.

## 1. Background

Despite its ancient origins and advances in treatment, cancer remains one of the most challenging medical problems worldwide. Surgery, chemotherapy, radiotherapy, and biological agents are used in cancer treatment (1). However, these approaches cannot cure or eradicate all cancers because of the complex nature and heterogeneity of cancer, serious adverse effects, and drug resistance. In some cases, chemoresistance is acquired during therapy, whereas in others, it is intrinsic and present before chemotherapy (2). Therefore, novel drugs are still needed for the treatment of different types of cancer as primary or adjuvant therapeutic agents, and many studies have focused on this issue.

Isocoumarins, a class of naturally occurring bicyclic lactones, possess diverse pharmacological activities (3, 4), including antidiabetic (3, 4), antibacterial (4), antifungal (4), antiviral (5), antiparasitic (4), antialgal (4), anti-inflammatory (4, 6), antioxidant (4, 7), and cytotoxic (4, 8-10) activities. These compounds are found in plants, fungi, bacterial strains, and even in insect pheromones and venom (11, 12). Their importance arises not only from their various biological effects but also from their use as intermediates in the synthesis of different heterocyclic structures (12).

## 2. Objectives

This study aimed to synthesize a series of novel ethyl 2-aminopyrano[3,2-c]isochromene-3-carboxylate derivatives using 4-hydroxyisocoumarin, aromatic aldehydes, and ethyl cyanoacetate in the presence of triethylamine, with ethanol as the reaction solvent. The reaction was performed as a one-pot, three-component process under reflux conditions. The cytotoxic effects of the synthesized compounds were subsequently evaluated in three cell lines: MCF-7 (breast cancer), A549 (lung cancer), and MCF-10A (normal human breast cells).

## 3. Methods

All chemicals and reagents were obtained from commercial sources. Dulbecco modified Eagle medium (DMEM) was purchased from Biosera (France), fetal bovine serum (FBS) from Gibco (USA), and MTT reagent from Melford (UK). Cell lines were obtained from the Iranian Biological Resource Center (IBRC) in Tehran, Iran. Melting points were determined using an Electrothermal 9100 apparatus without correction. IR spectra were recorded as KBr pellets on a Bruker Alpha FTIR spectrophotometer. ^1^H NMR (300 MHz) and ^13^C NMR (75 MHz) spectra were recorded in dimethyl sulfoxide (DMSO-d_6_) on a Bruker AVANCE III 300 MHz spectrometer using TMS as an internal standard. Coupling constants (J) are reported in hertz (Hz), and chemical shifts (δ) are reported in parts per million (ppm). Reactions were monitored by thin-layer chromatography (TLC) on aluminum-backed silica gel sheets (GF254). Spots were visualized under UV light at 254 nm. Elemental analyses were performed using a Heraeus CHN-O-Rapid analyzer.

### 3.1. General Procedure for the Preparation of Ethyl 2-Aminopyrano[3,2-C]isochromene-3-Carboxylate Derivatives (4a-O)

In a 50 mL round-bottom flask equipped with a magnetic stirring bar and reflux condenser, 1 mmol of 4-hydroxyisocoumarin (1) (13), 1.2 mmol of ethyl cyanoacetate (2), and 1 mmol of aromatic aldehydes (3a-o) were mixed with triethylamine (three drops) in 10 mL of ethanol. The mixture was stirred under reflux in an oil bath for two hours. Reaction progress was monitored by TLC using hexane/ethyl acetate as the mobile phase. After completion, the mixture was allowed to cool, and the formed product was collected by filtration. The product was washed with cold ethanol and recrystallized from ethanol to afford a pure solid for analysis.

Ethyl 2-amino-6-oxo-4-phenyl-4,6-dihydropyrano[3,2-c]isochromene-3-carboxylate (4a): Cream powder; yield: 88%; mp 229 - 231°C; IR (KBr): (ν_max_, cm^-1^) 3399 and 3290 (NH_2_), 3027 (CH, aromatic), 2983 (CH, aliphatic), 1738 and 1693 (2C=O), 1607 (C=C), 1094 (C-O); ^1^H NMR (300 MHz, DMSO) δ_ppm_: 8.14 (d, J=6 Hz, 1H, ArH), 8.04 - 7.99 (m, 1H, ArH), 7.86 (d, J=6 Hz, 1H, ArH), 7.80 (s, 2H, NH_2_), 7.71 - 7.66 (m, 1H, ArH), 7.35 - 7.20 (m, 5H, ArH), 4.73 (s, 1H, CH), 4.00 - 3.90 (m, 2H, OCH_2_CH_3_), 1.02 (t, J=6 Hz, 3H, OCH_2_CH_3_); ^13^C NMR (75 MHz, DMSO) δ_ppm_: 168.25 and 160.38 (2C=O), 159.90 (C-2), 144.19, 139.96, 136.07, 130.84, 130.07, 129.73, 128.77, 128.59, 128.34, 127.33, 120.35, 119.99, 76.26 (C-3), 59.30 (OCH_2_CH_3_), 31.15 (C-4), 14.55 (OCH_2_CH_3_); Anal. calcd. for C_21_H _17_NO_5_: C, 69.41; H, 4.72; N, 3.85%. Found: C, 69.32; H, 4.50; N, 3.69%.

Ethyl 2-amino-6-oxo-4-(p-tolyl)-4,6-dihydropyrano[3,2-c]isochromene-3-carboxylate (4b): White powder; yield: 91%; mp 219 - 221°C; IR (KBr): (ν_max_, cm^-1^) 3390 and 3276 (NH_2_), 3062 (CH, aromatic), 2979 (CH, aliphatic), 1720 and 1687 (2C=O), 1607 (C=C), 1098 (C-O); ^1^H NMR (300 MHz, DMSO) δ_ppm_: 8.14 (d, J=6 Hz, 1H, ArH), 8.03 - 7.98 (m, 1H, ArH), 7.85 (d, J=6 Hz, 1H, ArH), 7.77 (s, 2H, NH_2_), 7.70 - 7.65 (m, 1H, ArH), 7.18 - 7.10 (m, 4H, ArH), 4.68 (s, 1H, CH), 3.95 (q, J=6 Hz, 2H, OCH_2_CH_3_), 2.25 (s, 3H, CH_3_), 1.05 (t, J=6 Hz, 3H, OCH_2_CH_3_); ^13^C NMR (75 MHz, DMSO) δ_ppm_: 168.29 and 160.39 (2C=O), 159.85 (C-2), 141.23, 140.16, 136.42, 136.04, 130.86, 130.05, 129.67, 129.33, 128.49, 128.19, 120.32, 119.94, 76.37 (C-3), 59.31 (OCH_2_CH_3_), 39.88 (C-4), 21.08 (CH_3_), 14.59 (OCH_2_CH_3_); Anal. calcd. for C_22_H_19_NO_5_: C, 70.02; H, 5.07; N, 3.71%. Found: C, 69.88; H, 4.71; N, 3.48%.

Ethyl 2-amino-4-(4-fluorophenyl)-6-oxo-4,6-dihydropyrano[3,2-c]isochromene-3-carboxylate (4c): White powder; yield: 92%; mp 228 - 229°C; IR (KBr): (ν_max_, cm^-1^) 3391 and 3276 (NH_2_), 3068 (CH, aromatic), 2979 (CH, aliphatic), 1724 and 1689 (2C=O), 1606 (C=C), 1097 (C-O); ^1^H NMR (300 MHz, DMSO) δ_ppm_: 8.14 (d, J=6 Hz, 1H, ArH), 8.04 - 7.99 (m, 1H, ArH), 7.85 (d, J=6 Hz, 1H, ArH), 7.82 (s, 2H, NH_2_), 7.71 - 7.66 (m, 1H, ArH), 7.34 - 7.29 (m, 2H, ArH), 7.17 - 7.11 (m, 2H, ArH), 4.75 (s, 1H, CH), 4.02 - 3.90 (m, 2H, OCH_2_CH_3_), 1.02 (t, J=6 Hz, 3H, OCH_2_CH_3_); ^13^C NMR (75 MHz, DMSO) δ_ppm_: 168.18 and 163.17 (2C=O), 160.34 (C-2), 159.85, 140.38, 140.34, 139.60, 136.04, 130.80, 130.25, 130.14, 130.06, 129.77, 128.60, 120.38, 120.03, 115.63, 115.34, 76.10 (C-3), 59.31 (OCH_2_CH_3_), 39.59 (C-4), 14.56 (OCH_2_ CH_3_); Anal. calcd. for C_21_H _16_FNO_5_: C, 66.14; H, 4.23; N, 3.67%. Found: C, 65.87; H, 4.20; N, 3.47%.

Ethyl 2-amino-4-(4-bromophenyl)-6-oxo-4,6-dihydropyrano[3,2-c]isochromene-3-carboxylate (4d): White powder; yield: 89%; mp 227 - 228°C; IR (KBr): (ν_max_, cm^-1^) 3446 and 3318 (NH_2_), 3036 (CH, aromatic), 2979 (CH, aliphatic), 1725 and 1694 (2C=O), 1608 (C=C), 1091 (C-O); ^1^H NMR (300 MHz, DMSO) δ_ppm_: 8.13 (d, J=6 Hz, 1H, ArH), 8.00 (t, J=6 Hz, 1H, ArH), 7.84 (t, J=3 Hz, 3H, NH_2_, ArH), 7.67 (t, J=6 Hz, 1H, ArH), 7.50 (d, J=9 Hz, 2H, ArH), 7.24 (d, J=9 Hz, 2H, ArH), 4.72 (s, 1H, CH), 4.03 - 3.87 (m, 2H, OCH_2_CH_3_), 1.03 (t, J=6 Hz, 3H, OCH_2_ CH_3_); ^13^C NMR (75 MHz, DMSO) δ_ppm_: 168.10 and 160.27 (2C=O), 159.87 (C-2), 143.62, 139.25, 136.02, 131.64, 130.73, 130.61, 130.05, 129.79, 128.69, 120.39, 120.05, 75.75 (C-3), 59.37 (OCH_2_CH_3_), 39.84 (C-4), 14.57 (OCH_2_CH_3_); Anal. calcd. for C_21_H_16_BrNO_5_: C, 57.03; H, 3.65; N, 3.17%. Found: C, 56.71; H, 3.57; N, 2.91%.

Ethyl 2-amino-4-(4-nitrophenyl)-6-oxo-4,6-dihydropyrano[3,2-c]isochromene-3-carboxylate (4e): Pale yellow powder; yield: 88%; mp 218 - 219°C; IR (KBr): (ν_max_, cm^-1^) 3387 and 3278 (NH_2_), 3073 (CH, aromatic), 2987 (CH, aliphatic), 1729 and 1692 (2C=O), 1609 (C=C), 1522 and 1349 (NO_2_), 1096 (C-O); ^1^H NMR (300 MHz, DMSO) δ_ppm_: 8.21 - 8.17 (m, 2H, ArH), 8.14 (d, J=9 Hz, 1H, ArH), 8.05 - 8.00 (m, 1H, ArH), 7.92 (s, 2H, NH_2_), 7.86 (d, J=6 Hz, 1H, ArH), 7.72 - 7.67 (m, 1H, ArH), 7.61 - 7.57 (m, 2H, ArH), 4.92 (s, 1H, CH), 3.99 - 3.89 (m, 2H, OCH_2_CH_3_), 1.01 (t, J=6 Hz, 3H, OCH_2_CH_3_); ^13^C NMR (75 MHz, DMSO) δ_ppm_: 167.93 and 160.17 (2C=O), 159.92 (C-2), 151.78, 146.95, 138.47, 136.08, 130.62, 130.08, 129.99, 129.80, 129.01, 124.03, 120.48, 120.17, 75.21 (C-3), 59.45 (OCH_2_CH_3_), 40.28 (C-4), 14.53 (OCH_2_CH_3_); Anal. calcd. for C_21_H _16_N_2_O_7_: C, 61.77; H, 3.95; N, 6.86%. Found: C, 61.69; H, 3.80; N, 6.77%.

Ethyl 2-amino-6-oxo-4-(m-tolyl)-4,6-dihydropyrano[3,2-c]isochromene-3-carboxylate (4f): White powder; yield: 91%; mp 221 - 222°C; IR (KBr): (ν_max_, cm^-1^) 3413 and 3296 (NH_2_), 3014 (CH, aromatic), 2987 (CH, aliphatic), 1732 and 1690 (2C=O), 1607 (C=C), 1098 (C-O); ^1^H NMR (300 MHz, DMSO) δ_ppm_: 8.13 (d, J=9 Hz, 1H, ArH), 8.02 - 7.97 (m, 1H, ArH), 7.85 (d, J=9 Hz, 1H, ArH), 7.78 (s, 2H, NH_2_), 7.69 - 7.63 (m, 1H, ArH), 7.19 (t, J=9 Hz, 1H, ArH), 7.08 - 7.01 (m, 3H, ArH), 4.68 (s, 1H, CH), 4.01 - 3.90 (m, 2H, OCH_2_CH_3_), 2.27 (s, 3H, CH_3_), 1.04 (t, J=6 Hz, 3H, OCH_2_CH_3_); ^13^C NMR (75 MHz, DMSO) δ_ppm_: 168.28 and 160.39 (2C=O), 159.83 (C-2), 144.13, 140.03, 137.78, 136.00, 130.85, 130.03, 129.65, 128.92, 128.62, 128.00, 125.57, 120.34, 119.97, 76.38 (C-3), 59.29 (OCH_2_CH_3_), 40.28 (C-4), 21.48 (CH_3_), 14.52 (OCH_2_CH_3_); Anal. calcd. for C_22_H_19_NO_5_: C, 70.02; H, 5.07; N, 3.71%. Found: C, 69.78; H, 3.85; N, 3.46%.

Ethyl 2-amino-4-(3-methoxyphenyl)-6-oxo-4,6-dihydropyrano[3,2-c]isochromene-3-carboxylate (4g): White powder; yield: 90%; mp 196 - 197 °C; IR (KBr): (ν_max_, cm^-1^) 3392 and 3275 (NH_2_), 3046 (CH, aromatic), 2980 (CH, aliphatic), 1724 and 1690 (2C=O), 1607 (C=C), 1100 (C-O); ^1^H NMR (300 MHz, DMSO) δ_ppm_: 8.14 (d, J=6 Hz, 1H, ArH), 8.03 - 7.98 (m, 1H, ArH), 7.85 (d, J=6 Hz, 1H, ArH), 7.80 (s, 2H, NH_2_), 7.70 - 7.64 (m, 1H, ArH), 7.26 - 7.21 (m, 1H, ArH), 6.86 - 6.79 (m, 3H, ArH), 4.70 (s, 1H, CH), 4.02 - 3.92 (m, 2H, OCH_2_CH_3_), 3.93 (s, 3H, OCH_3_), 1.05 (t, J=6 Hz, 3H, OCH_2_CH_3_); ^13^C NMR (75 MHz, DMSO) δ_ppm_: 168.26 and 160.37 (2C=O), 159.90 (C-2), 159.61, 145.76, 139.86, 136.02, 130.82, 130.05, 129.87, 129.69, 128.62, 120.45, 120.35, 119.99, 114.56, 112.26, 76.18 (C-3), 59.32 (OCH_2_CH_3_), 55.42 (OCH_3_), 40.28 (C-4), 14.58 (OCH_2_CH_3_); Anal. calcd. for C_22_H_19_NO_6_: C, 67.17; H, 4.87; N, 3.56%. Found: C, 66.88; H, 4.60; N, 3.41%.

Ethyl 2-amino-4-(3-fluorophenyl)-6-oxo-4,6-dihydropyrano[3,2-c]isochromene-3-carboxylate (4h): White powder; yield: 88%; mp 216 - 217°C; IR (KBr): (ν_max_, cm^-1^) 3405 and 3290 (NH_2_), 3041 (CH, aromatic), 2978 (CH, aliphatic), 1730 and 1690 (2C=O), 1609 (C=C), 1098 (C-O); ^1^H NMR (300 MHz, DMSO) δ_ppm_: 8.14 (d, J=6 Hz, 1H, ArH), 8.01 (t, J=6 Hz, 1H, ArH), 7.85 (t, J=6 Hz, 3H, NH_2_, ArH), 7.68 (t, J=6 Hz, 1H, ArH), 7.36 (q, J=6 Hz, 1H, ArH), 7.15 - 7.04 (m, 3H, ArH), 4.77 (s, 1H, CH), 4.05 - 3.87 (m, 2H, OCH_2_CH_3_), 1.01 (t, J=6 Hz, 3H, OCH_2_CH_3_); ^13^C NMR (75 MHz, DMSO) δ_ppm_: 168.12 and 164.17 (2C=O), 160.94 (C-2), 160.29, 159.91, 147.14, 147.06, 139.13, 136.01, 130.75, 130.60, 130.04, 129.79, 128.77, 124.41, 124.38, 120.42, 120.10, 115.32, 115.04, 114.30, 114.02, 75.71 (C-3), 59.32 (OCH_2_CH_3_), 40.05 (C-4), 14.51 (OCH_2_CH_3_); Anal. calcd. for C_21_H _16_FNO_5_: C, 66.14; H, 4.23; N, 3.67%. Found: C, 65.87; H, 4.11; N, 3.44%.

Ethyl 2-amino-4-(3-chlorophenyl)-6-oxo-4,6-dihydropyrano[3,2-c]isochromene-3-carboxylate (4i): White powder; yield: 90%; mp 211 - 212°C; IR (KBr): (ν_max_, cm^-1^) 3415 and 3303 (NH_2_), 3059 (CH, aromatic), 2977 (CH, aliphatic), 1732 and 1692 (2C=O), 1614 (C=C), 1097 (C-O); ^1^H NMR (300 MHz, DMSO) δ_ppm_: 8.14 (d, J=6 Hz, 1H, ArH), 8.04 - 7.99 (m, 1H, ArH), 7.86 (t, J=6 Hz, 3H, NH_2_, ArH), 7.71 - 7.66 (m, 1H, ArH), 7.38 - 7.23 (m, 4H, ArH), 4.76 (s, 1H, CH), 4.05 - 3.86 (m, 2H, OCH_2_CH_3_), 1.02 (t, J=6 Hz, 3H, OCH_2_CH_3_); ^13^C NMR (75 MHz, DMSO) δ_ppm_: 168.07 and 160.29 (2C=O), 159.87 (C-2), 146.66, 139.01, 136.03, 133.29, 130.70, 130.06, 129.84, 128.84, 128.36, 127.35, 127.10, 120.44, 120.13, 75.66 (C-3), 59.36 (OCH_2_CH_3_), 40.07 (C-4), 14.50 (OCH_2_CH_3_); Anal. calcd. for C_21_H _16_ClNO_5_: C, 63.40; H, 4.05; N, 3.52%. Found: C, 63.22; H, 3.75; N, 3.36%.

Ethyl 2-amino-4-(3-bromophenyl)-6-oxo-4,6-dihydropyrano[3,2-c]isochromene-3-carboxylate (4j): White powder; yield: 91%; mp 206 - 207°C; IR (KBr): (ν_max_, cm^-1^) 3414 and 3302 (NH_2_), 3057 (CH, aromatic), 2974 (CH, aliphatic), 1731 and 1692 (2C=O), 1614 (C=C), 1097 (C-O); ^1^H NMR (300 MHz, DMSO) δ_ppm_: 8.14 (d, J=6 Hz, 1H, ArH), 8.05 - 7.99 (m, 1H, ArH), 7.85 (t, J=3 Hz, 3H, NH_2_, ArH), 7.72 - 7.66 (m, 1H, ArH), 7.46 - 7.40 (m, 2H, ArH), 7.32 - 7.25 (m, 2H, ArH), 4.75 (s, 1H, CH), 4.03 - 3.89 (m, 2H, OCH_2_CH_3_), 1.03 (t, J=6 Hz, 3H, OCH_2_ CH_3_); ^13^C NMR (75 MHz, DMSO) δ_ppm_: 168.06 and 160.30 (2C=O), 159.85 (C-2), 146.90, 139.01, 136.04, 131.27, 131.04, 130.72, 130.25, 130.07, 129.86, 128.86, 127.48, 121.91, 120.45, 120.14, 75.70 (C-3), 59.37 (OCH_2_CH_3_), 39.73 (C-4), 14.51 (OCH_2_CH_3_); Anal. calcd. for C_21_H _16_BrNO_5_: C, 57.03; H, 3.65; N, 3.17%. Found: C, 56.71; H, 3.22; N, 2.91%.

Ethyl 2-amino-4-(3-nitrophenyl)-6-oxo-4,6-dihydropyrano[3,2-c]isochromene-3-carboxylate (4k): White powder; yield: 87%; mp 208 - 209°C; IR (KBr): (ν_max_, cm^-1^) 3383 and 3274 (NH_2_), 3061 (CH, aromatic), 2986 (CH, aliphatic), 1729 and 1689 (2C=O), 1614 (C=C), 1092 (C-O), 1528 and 1348 (NO_2_); ^1^H NMR (300 MHz, DMSO) δ_ppm_: 8.14 - 8.10 (m, 3H, ArH), 8.05 - 8.00 (m, 1H, ArH), 7.92 (s, 2H, NH_2_), 7.87 (d, J=6 Hz, 1H, ArH), 7.80 - 7.77 (m, 1H, ArH), 7.72 - 7.60 (m, 2H, ArH), 4.94 (s, 1H, CH), 4.00 - 3.88 (m, 2H, OCH_2_CH_3_), 1.00 (t, J=6 Hz, 3H, OCH_2_CH_3_); ^13^C NMR (75 MHz, DMSO) δ_ppm_: 167.94 and 160.22 (2C=O), 159.91 (C-2), 148.17, 146.42, 138.48, 136.03, 135.25, 130.64, 130.36, 130.05, 129.94, 129.11, 123.08, 122.49, 120.49, 120.25, 75.41 (C-3), 59.43 (OCH_2_CH_3_), 40.08 (C-4), 14.45 (OCH_2_CH_3_); Anal. calcd. for C_21_H _16_N_2_O_7_: C, 61.77; H, 3.95; N, 6.86%. Found: C, 61.37; H, 3.60; N, 6.45%.

Ethyl 2-amino-6-oxo-4-(o-tolyl)-4,6-dihydropyrano[3,2-c]isochromene-3-carboxylate (4l): Yellow powder; yield: 88%; mp 234 - 235°C; IR (KBr): (ν_max_, cm^-1^) 3386 and 3277 (NH_2_), 3018 (CH, aromatic), 2975 (CH, aliphatic), 1721 and 1687 (2C=O), 1615 (C=C), 1091 (C-O); ^1^H NMR (300 MHz, DMSO) δ_ppm_: 8.11 (d, J=6 Hz, 1H, ArH), 8.02 - 7.97 (m, 1H, ArH), 7.83 (d, J=6 Hz, 1H, ArH), 7.79 (s, 2H, NH_2_), 7.68 - 7.63 (m, 1H, ArH), 7.17 - 7.05 (m, 4H, ArH), 5.02 (s, 1H, CH), 3.89 (q, J=6 Hz, 2H, OCH_2_CH_3_), 2.53 (s, 3H, CH_3_), 0.94 (t, J=6 Hz, 3H, OCH_2_CH_3_); ^13^C NMR (75 MHz, DMSO) δ_ppm_: 168.25 and 160.27 (2C=O), 160.07 (C-2), 143.27, 140.77, 136.17, 136.02, 130.84, 130.26, 130.03, 129.62, 128.16, 126.98, 126.86, 120.26, 119.91, 76.38 (C-3), 59.29 (OCH_2_CH_3_), 35.83 (C-4), 19.60 (CH_3_), 14.39 (OCH_2_CH_3_); Anal. calcd. for C_22_H_19_NO_5_: C, 70.02; H, 5.07; N, 3.71%. Found: C, 69.81; H, 4.93; N, 3.56%.

Ethyl 2-amino-4-(2-fluorophenyl)-6-oxo-4,6-dihydropyrano[3,2-c]isochromene-3-carboxylate (4m): White powder; yield: 89%; mp 238 - 239°C; IR (KBr): (ν_max_, cm^-1^) 3402 and 3287 (NH_2_), 3042 (CH, aromatic), 2973 (CH, aliphatic), 1735 and 1693 (C=O), 1606 (C=C), 1096 (C-O); ^1^H NMR (300 MHz, DMSO) δ_ppm_: 8.13 (d, J=6 Hz, 1H, ArH), 8.03 - 7.98 (m, 1H, ArH), 7.84 (t, J=6 Hz, 3H, NH_2_, ArH), 7.70 - 7.64 (m, 1H, ArH), 7.31 - 7.24 (m, 2H, ArH), 7.18 - 7.12 (m, 2H, ArH), 5.06 (s, 1H, CH), 3.98 - 3.86 (m, 2H, OCH_2_CH_3_), 0.98 (t, J=6 Hz, 3H, OCH_2_CH_3_); ^13^C NMR (75 MHz, DMSO) δ_ppm_: 168.08 and 162.35 (2C=O), 160.27 (C-2), 160.15, 159.09, 138.75, 136.03, 131.08, 130.91, 130.72, 130.64, 130.59, 130.05, 129.78, 129.38, 129.27, 128.91, 124.96, 124.92, 120.33, 120.08, 115.79, 115.50, 74.98 (C-3), 59.28 (OCH_2_CH_3_), 33.84 (C-4), 14.33 (OCH_2_CH_3_); Anal. calcd. for C_21_H _16_FNO_5_: C, 66.14; H, 4.23; N, 3.67%. Found: C, 65.80; H, 3.99; N, 3.51%.

Ethyl 2-amino-4-(2-chlorophenyl)-6-oxo-4,6-dihydropyrano[3,2-c]isochromene-3-carboxylate (4n): White powder; yield: 91%; mp 240 - 241°C; IR (KBr): (ν_max_, cm^-1^) 3400 and 3287 (NH_2_), 3061 (CH, aromatic), 2975 (CH, aliphatic), 1731 and 1677 (C=O), 1607 (C=C), 1093 (C-O); ^1^H NMR (300 MHz, DMSO) δ_ppm_: 8.13 (d, J=6 Hz, 1H, ArH), 8.04 - 7.98 (m, 1H, ArH), 7.85 (d, J=6 Hz, 3H, NH_2_, ArH), 7.70 - 7.65 (m, 1H, ArH), 7.43 - 7.40 (m, 1H, ArH), 7.32 - 7.21 (m, 3H, ArH), 5.29 (s, 1H, CH), 3.95 - 3.84 (m, 2H, OCH_2_CH_3_), 0.95 (t, J=6 Hz, 3H, OCH_2_CH_3_); ^13^C NMR (75 MHz, DMSO) δ_ppm_: 168.12 and 160.21 (2C=O), 160.13 (C-2), 141.62, 139.03, 136.02, 133.36, 130.83, 130.69, 130.05, 129.81, 129.64, 128.95, 128.75, 127.95, 120.38, 120.12, 75.45 (C-3), 59.27 (OCH_2_CH_3_), 37.21 (C-4), 14.36 (OCH_2_CH_3_); Anal. calcd. for C_21_H _16_ClNO_5_: C, 63.40; H, 4.05; N, 3.52%. Found: C, 63.27; H, 3.82; N, 3.22%.

Ethyl 2-amino-4-(2,4-dichlorophenyl)-6-oxo-4,6-dihydropyrano[3,2-c]isochromene-3-carboxylate (4o): Cream powder; yield: 90%; mp 212 - 214°C; IR (KBr): (ν_max_, cm^-1^) 3402 and 3289 (NH_2_), 3062 (CH, aromatic), 2981 (CH, aliphatic), 1723 and 1694 (2C=O), 1615 (C=C), 1097 (C-O); ^1^H NMR (300 MHz, DMSO) δ_ppm_: 8.14 (d, J=6 Hz, 1H, ArH), 8.05 - 8.00 (m, 1H, ArH), 7.90 (s, 2H, NH_2_), 7.85 (d, J=6 Hz, 1H, ArH), 7.72 - 7.67 (m, 1H, ArH), 7.57 (d, J=6 Hz, 1H, ArH), 7.39 - 7.32 (m, 2H, ArH), 5.28 (s, 1H, CH), 3.97 - 3.86 (m, 2H, OCH_2_CH_3_), 0.98 (t, J=6 Hz, 3H, OCH_2_CH_3_); ^13^C NMR (75 MHz, DMSO) δ_ppm_: 167.97 and 160.14 (2C=O), 140.84 (C-2), 138.47, 136.06, 134.23, 132.50, 132.19, 130.62, 130.08, 129.93, 128.94, 128.88, 128.21, 120.42, 120.20, 75.02 (C-3), 59.36 (OCH_2_CH_3_), 36.86 (C-4), 14.42 (OCH_2_CH_3_); Anal. calcd. for C_21_H_15_Cl_2_NO_5_: C, 58.35; H, 3.50; N, 3.24%. Found: C, 58.13; H, 3.34; N, 3.01%.

### 3.2. Cell Culture

The MCF-7 (IBRC C10082), A549 (IBRC C10080), and MCF-10A (IBRC C10788) cell lines were cultured in DMEM supplemented with 10% heat-inactivated FBS, 100 U/mL penicillin, and 100 μg/mL streptomycin. Cultures were incubated in a humidified atmosphere containing 5% CO_2_ at 37 °C.

### 3.3. Cytotoxicity Assay

The MTT assay was performed to assess the cytotoxicity of the compounds. When cells reached 80% confluency, they were trypsinized, counted, and seeded in 96-well microplates at a density of 1 × 10^4^ cells per well. The plates were incubated under the conditions described above for 24 hours. The next day, the growth medium in each well was replaced with fresh medium containing different concentrations of the compounds (7.81 to 500 μg/mL) or doxorubicin (2 to 32 μg/mL), which was used as the standard drug because it is a well-documented anticancer agent with activity against a wide range of cancer cell lines. The microplates were then incubated for 24 hours. The compounds were dissolved in the minimum amount of DMSO at high concentration and diluted with FBS-free medium; thus, the solvent concentration at the highest tested concentration (500 μg/mL) was below 1% (v/v). A solvent control was also used for each concentration. On the third day, 10 μL of MTT dye (5 mg/mL) was added to each well, and the microplates were incubated for 3 hours in the dark. Then, 100 μL of DMSO was added to each well to dissolve the formazan crystals formed by viable cells. Absorbance was measured at 570 nm with a reference wavelength of 620 nm using a multiwell plate reader (14). The absorbance values were used to calculate the percentage of viable cells. IC_50_ values were determined using GraphPad Prism version 5 (San Diego, CA, USA), with a nonlinear dose-response curve generated from the percentage of viable cells against the logarithm of compound concentrations. The SIs were calculated by dividing the IC_50_ value of each compound for MCF-10A cells by the IC_50_ value of the same compound for A549 or MCF-7 cells (15). Differences between compound concentrations were compared using one-way ANOVA with a Tukey post hoc test, with P < 0.05 considered statistically significant.

## 4. Results

**Table 1.** Selected Physicochemical Descriptors of Representative Compounds Calculated Using Standard Fragments

Compound	Substituent	MW (g/mol)	cLogP (est.)	HBD	HBA	TPSA (Å^2^)
**4a**	H	~363	~2.2	1	6	~86
**4c**	4-fluoro	~381	~2.3	1	6	~86
**4g**	3-methoxy	~393	~2.5	1	7	~95
**4m**	2-fluoro	~381	~2.4	1	6	~86
**4n**	2-chloro	~397	~2.8	1	6	~86
**4o**	2,4-dichloro	~432	~3.2	1	6	~86

### 4.1. Chemistry

**Figure 1.**
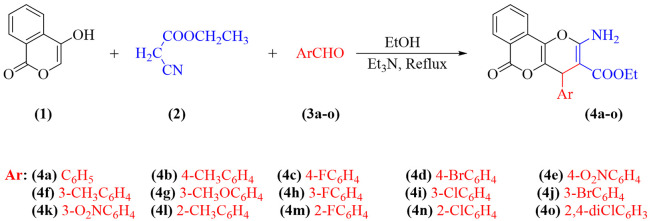
Synthesis of ethyl 2-aminopyrano[3,2-c]isochromene-3-carboxylate derivatives (4a-o)

A series of novel ethyl 2-aminopyrano[3,2-c]isochromene-3-carboxylate derivatives (4a-o) were synthesized as shown in Figure 1. Building on our previous work on environmentally friendly multicomponent reactions (16-18), we performed a one-pot, three-component reaction of 4-hydroxyisocoumarin (1) (13), ethyl cyanoacetate (2), and aromatic aldehydes (3a-o) in the presence of triethylamine, using ethanol as the solvent, under reflux conditions. After completion, the crude product was purified by recrystallization to afford a series of new ethyl 2-amino-4-aryl-4,6-dihydropyrano[3,2-c]isochromene-3-carboxylate derivatives (4a-o) in 87% to 92% yield. The high yields, operational simplicity, clean reaction conditions, and facile product isolation are advantages of this procedure, making it a practical method for synthesizing these compounds.

A series of substituted benzaldehyde derivatives bearing electron-donating (CH_3_ and OCH_3_) and electron-withdrawing (NO_2_, F, Cl, and Br) groups at the para (4b-4e), meta (4f-4k), ortho (4l-4n), and disubstituted (4o) positions were synthesized. In total, 15 derivatives of ethyl 2-aminopyrano[3,2-c]isochromene-3-carboxylate (4a-o) were successfully synthesized, and their structures were comprehensively characterized by standard spectroscopic techniques (IR, ^1^H NMR, and ^13^C NMR) and elemental analysis.

### 4.2. Cytotoxicity Assay

**Figure 2.**
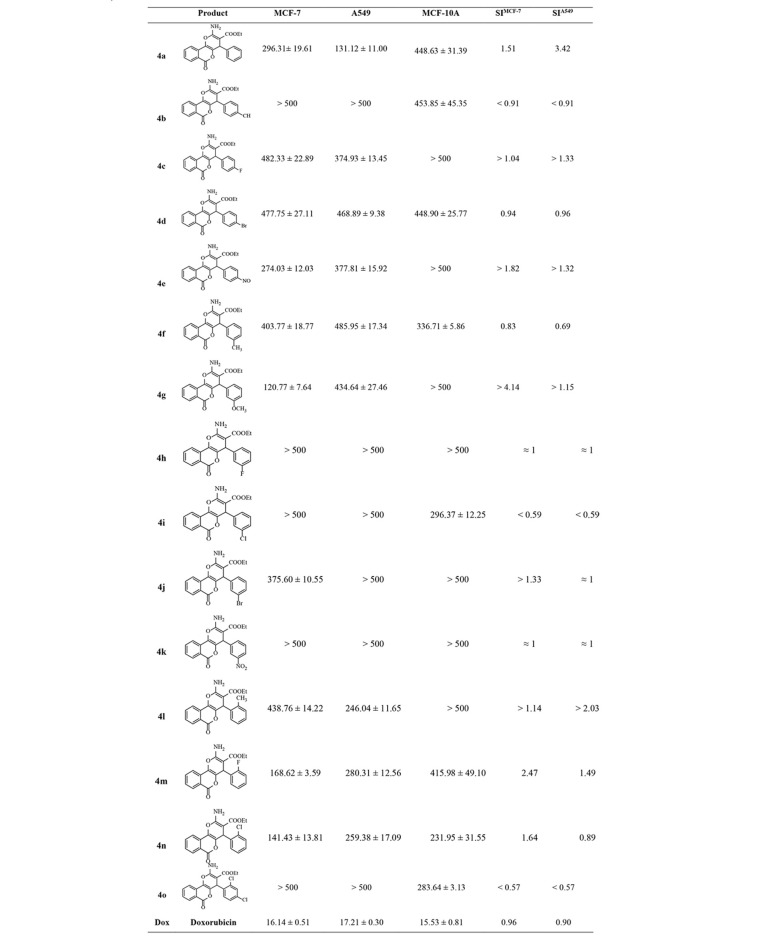
The IC50 (μg/mL) values (Mean ± S.E.M) of the synthesized compounds tested on MCF-7, A549, MCF-10A, and 3T3 cell lines, and the calculated SI values

The cytotoxic activity of the synthesized derivatives was evaluated using the MTT assay against two cancer cell lines (MCF-7 and A549) and one normal cell line (MCF-10A). As summarized in Figure 2, the most active compounds against MCF-7 cells were 4g, 4n, and 4m, with IC_50_ values of 120.77 ± 7.64, 141.43 ± 13.81, and 168.62 ± 3.59 μg/mL, respectively. Against A549 cells, compounds 4a, 4l, and 4n showed comparatively lower IC_50_ values (131.12 - 259.38 μg/mL). Most other derivatives had IC_50_ values above 300 μg/mL.

**Figure 3.**
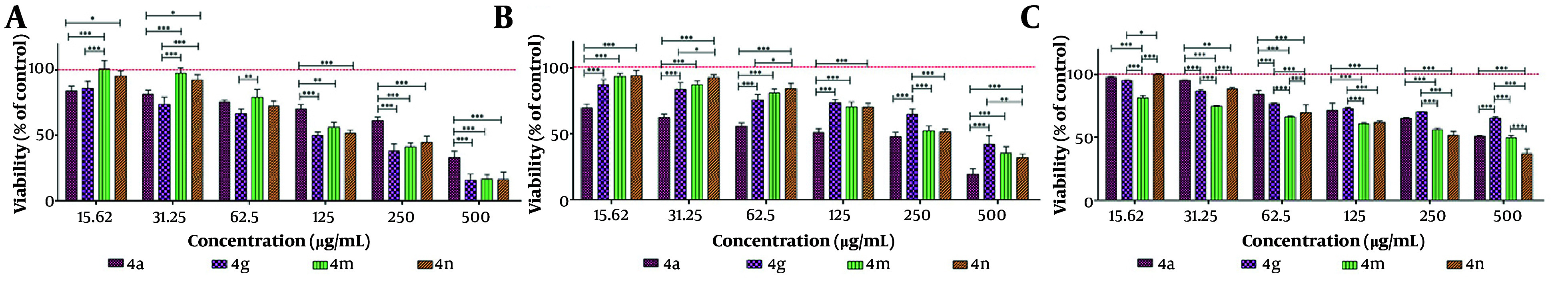
Viability percentages of (A) MCF-7; (B) A549; and (C) MCF-10A cells treated with various concentrations of selected synthesized compounds for 24 hours, as assessed by the MTT assay. For clarity, not all tested compounds and concentrations are displayed in the graph. All data were normalized to the solvent control (100%), indicated by the horizontal red dashed line. Bars represent mean ± SD (n ≥ 3). Values slightly exceeding 100% reflect normalization to the solvent control and normal experimental variability. ***P < 0.001, **P < 0.01, and *P < 0.05.

**Figure 4.**
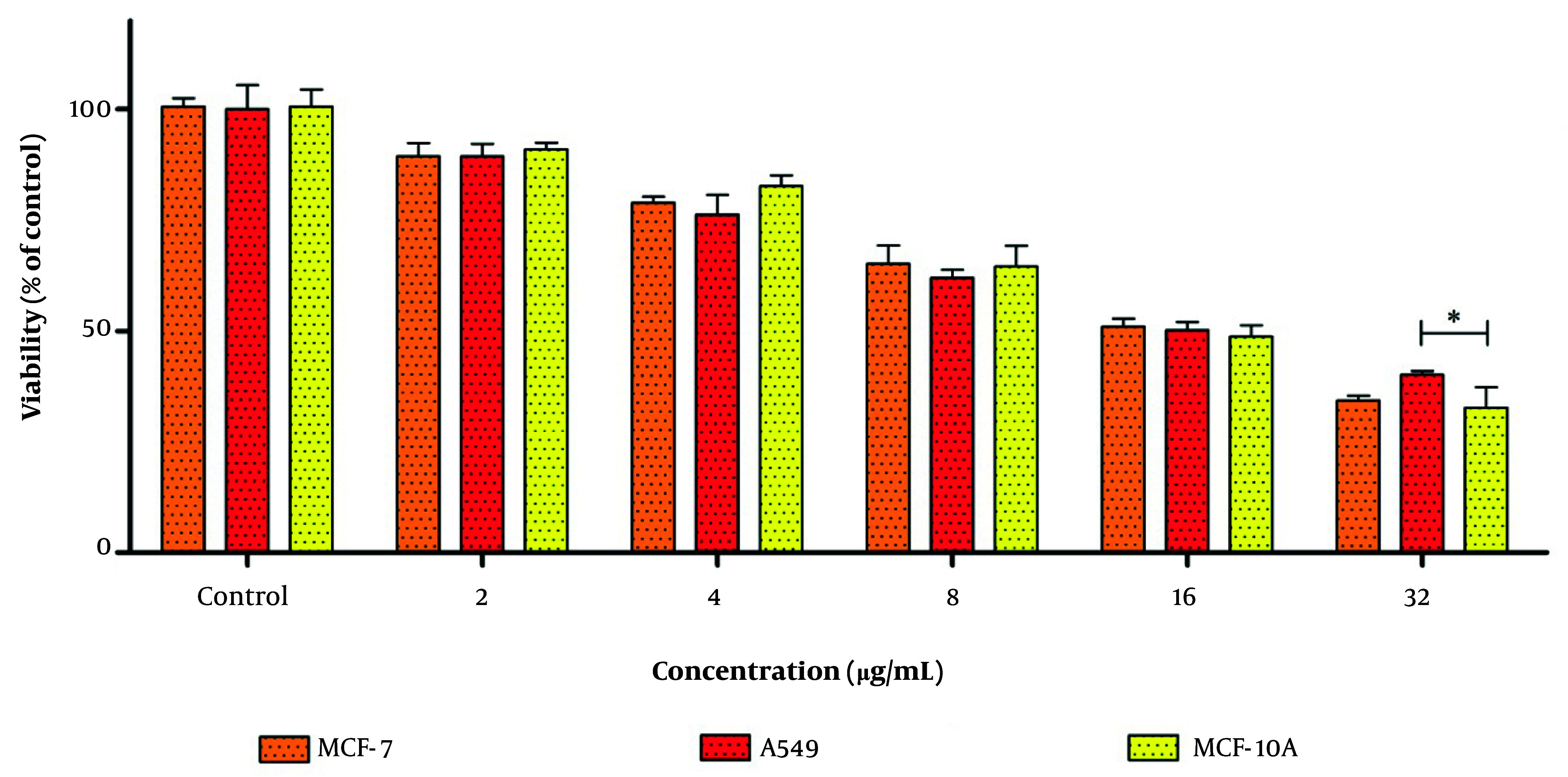
Viability percentages of MCF-7, A549, and MCF-10A cells treated with various concentrations of doxorubicin for 24 hours, as assessed by the MTT assay. Bars represent mean ± SD (n ≥ 3). *P < 0.05.

According to the National Cancer Institute (NCI) classification, compounds with IC_50_ values lower than 20 μg/mL are considered highly cytotoxic, those between 21 and 200 μg/mL are considered moderately cytotoxic, those between 201 and 500 μg/mL are considered weakly cytotoxic, and those greater than 500 μg/mL are considered non-cytotoxic (19). Based on this classification, most synthesized compounds were in the weak-to-moderate cytotoxicity range. Several derivatives (4b, 4h, 4i, 4j, 4k, and 4o) had IC_50_ values above 500 μg/mL and were therefore considered non-cytotoxic under these conditions. Most compounds showed mild or no toxicity toward normal MCF-10A cells (Figure 3). Doxorubicin, used as a reference drug, had substantially lower IC_50_ values under identical experimental conditions, confirming the relatively limited potency of the synthesized derivatives (Figure 4).

Although the observed cytotoxic activity was limited, these results provide preliminary insight into the biological profile of this scaffold and may guide future structural optimization.

**Figure 5.**
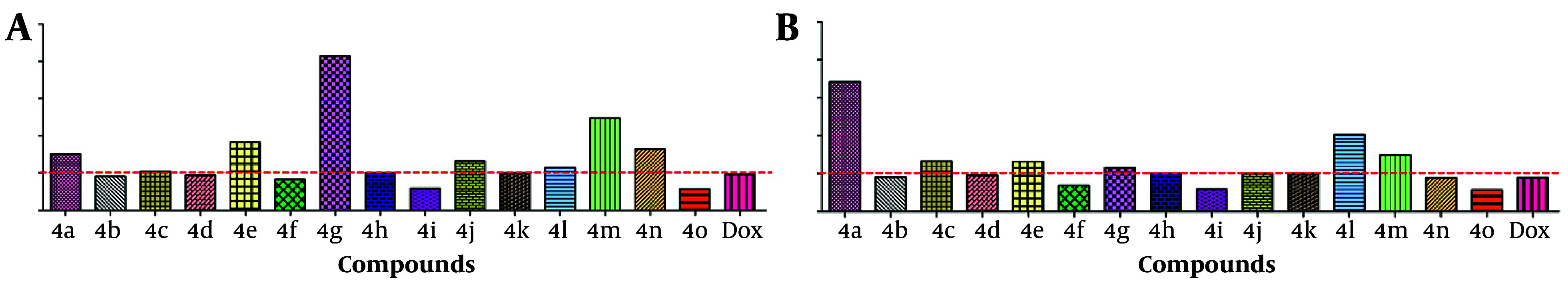
The SI values of the tested compounds for A, MCF-7 and B, A549 cells calculated by dividing the IC50 value of the compound for normal cell to the IC_50_ value of the compound for the cancerous cell. The horizontal red dashed line is used for comparison to the SI of Dox (Dox.: doxorubicin).

The SI values were calculated to estimate preferential toxicity toward cancer cells relative to normal cells. Although some compounds showed SI values greater than 1, most remained below the generally accepted threshold for meaningful selectivity (SI ≥ 3 - 10) (20, 21). Therefore, the observed selectivity should be interpreted cautiously. Overall, the synthesized derivatives showed limited potency and modest selectivity compared with doxorubicin (Figure 5).

Taken together, these findings indicate that although certain structural features, such as ortho-halogen substitution, may influence cytotoxic activity, the current scaffold demonstrates only weak-to-moderate biological effects under the tested conditions. Further structural refinement is required to enhance both potency and selectivity before advanced biological evaluation.

## 5. Discussion

A detailed structure–activity relationship (SAR) analysis suggests that both the nature and position of substituents on the phenyl ring markedly influence cytotoxic activity within this series. A positional dependence was observed among halogenated derivatives. Ortho-substituted compounds generally exhibited lower IC_50_ values than their meta- and para-substituted counterparts. For example, the ortho-fluoro derivative 4m showed a substantially lower IC_50_ value against MCF-7 cells (168.62 μg/mL) than the para-fluoro analogue 4c (482.30 μg/mL), indicating an approximately threefold enhancement in activity. A similar trend was observed for the ortho-chloro compound 4n, which displayed improved potency relative to the meta- and para-chloro derivatives (4i and 4d, respectively). This positional effect may be associated with steric and electronic influences that alter molecular conformation or cellular interactions. However, given the overall moderate activity levels, these observations should be considered preliminary.

In contrast, para-substituted derivatives consistently showed reduced activity, suggesting that excessive planarity or an unfavorable electronic distribution may impair effective target engagement. Introducing a chlorine atom at the para position in addition to the ortho position, as shown by comparison of compound 4o with 4n, rendered the molecule non-toxic. The IC_50_ values of compound 4n were 141.43 ± 13.81 and 259.38 ± 17.09 μg/mL for MCF-7 and A549 cells, respectively, and these values increased by more than 3.5-fold and 2-fold, respectively, after the addition of the second Cl atom in compound 4o. In contrast, another study synthesized halogenated dihydropyrano[3,2-b]chromene-3-carbonitrile derivatives and evaluated their cytotoxicity in the MCF-7 cell line, concluding that cytotoxicity increased when a group was present at the para position of the aromatic ring (22).

The electronic nature of the substituents also influenced cytotoxicity. Electron-donating substituents, such as the meta-methoxy group, were associated with comparatively improved activity in some cases (4g), whereas strong electron-withdrawing groups, such as nitro, especially when positioned at the meta or para locations (e.g., 4k and 4e), generally resulted in weak or absent cytotoxicity.

Disubstitution on the phenyl ring, as observed in compound 4o, led to a complete loss of cytotoxic activity, likely due to excessive steric hindrance that interferes with productive molecular interactions. Additionally, bulky substituents at unfavorable positions may negatively affect membrane permeability or intracellular distribution.

Although certain derivatives showed relatively higher SI values than other compounds in this series, their selectivity remains limited in absolute terms. Therefore, these compounds cannot be considered selective anticancer agents at this stage, but they may provide structural insight for further optimization.

Overall, the SAR trends suggest that small ortho substitution and balanced lipophilicity may modestly improve activity within this scaffold (Table 1). Nevertheless, substantial structural modification would be necessary to achieve biologically meaningful potency.

Although the observed cytotoxic activity was limited, the present findings contribute to the growing body of literature on biologically active isocoumarin-based scaffolds. The present study was limited to a preliminary in vitro MTT assay performed in only two cancer cell lines. No mechanistic assays were performed, and the molecular targets remain unidentified. Therefore, the current results should be interpreted as an initial biological characterization of these derivatives.

### 5.1. Conclusions

In summary, this study describes the efficient synthesis of a new series of ethyl 2-amino-4-aryl-4,6-dihydropyrano[3,2-c]isochromene-3-carboxylate derivatives via a one-pot, three-component reaction. The compounds were obtained in high yields and fully characterized by spectroscopic techniques, demonstrating the practicality of this synthetic approach. The advantages of this procedure include simple operational steps, clean reaction conditions, and easy product isolation. Biological evaluation revealed weak to moderate cytotoxic activity against the MCF-7 and A549 cancer cell lines, with limited selectivity relative to normal MCF-10A cells. Ortho substitution on the phenyl ring was associated with relatively improved activity within this series; however, overall potency remained substantially lower than that of the reference drug doxorubicin. These findings indicate that, although the synthesized scaffold exhibits measurable biological activity, it cannot currently be considered a promising anticancer candidate in its present structural form. Further structural optimization would be necessary to enhance cytotoxic potency and selectivity before advanced mechanistic or in-depth biological investigations.

## Data Availability

The data presented in this study are uploaded in this manuscript and are openly available for readers upon request.
